# Phenotypic, chemical component and molecular assessment of genetic diversity and population structure of *Morinda officinalis* germplasm

**DOI:** 10.1186/s12864-022-08817-w

**Published:** 2022-08-19

**Authors:** Zhenhua Luo, Zien Chen, Mengyun Liu, Li Yang, Zhimin Zhao, Depo Yang, Ping Ding

**Affiliations:** 1 School of Pharmaceutical Sciences, Guangzhou University of Chinese Medicine, Guangzhou, 510006 China; 2 School of Pharmacy, Sun Yat-sen University, Guangzhou, 510006 China

**Keywords:** *Morinda officinalis*, Single-nucleotide polymorphism, Genetic diversity, Morphological traits, Chemical components

## Abstract

**Background:**

*Morinda officinalis* How (MO) is a perennial herb distributed in tropical and subtropical regions, which known as one of the “Four Southern Herbal Medicines”. The extent of genetic variability and the population structure of MO are presently little understood. Here, nine morphological traits, six chemical components and Single nucleotide polymorphism (SNP) markers were used in integrative research of MO germplasm variation among 88 individuals collected from ten populations across four geographical provinces of China.

**Results:**

Both phenotype and chemical composition have significant genetic variation, and there is a certain correlation between them such as root diameter and the nystose content, as well as geographical distribution. The principal component analysis (PCA) showed the leaf length, leaf width, nystose, 1^F^-furanosaccharide nystose, and the section color were the major contributors to diversity. The cluster analysis based on phenotypic and oligosaccharide data distinguished three significant groups, which was consistent with the result of a corresponding analysis with 228,615 SNP markers, and importantly, they all showed a significant correlation with geographical origin. However, there was little similarity between two cluster results. The Shannon’s information index (I) varied from 0.17 to 0.53 with a mean of 0.37, suggesting a high level of genetic diversity in MO populations, which mainly existed among individuals within populations, accounting for 99.66% of the total according to the analysis of molecular variance (AMOVA) results. Each population also maintains the connection because of certain gene communication, so that the genetic differentiation between populations was not very significant. The STRUCTURE software was used to analyse the population structure and the result showed that 88 accessions were clustered into three groups, and 67% of them were pure type, which was also confirmed through PCA.

**Conclusions:**

The comprehensive study of phenotypic, chemical and molecular markers will provide valuable information for future breeding plans and understanding the phylogenetic relationship of MO population.

**Supplementary Information:**

The online version contains supplementary material available at 10.1186/s12864-022-08817-w.

## Introduction

*Morinda officinalis* How (MO) is a monoecious, perennial vine, widely distributed in tropical and subtropical regions. It is native to the mountainous regions of southern in China such as Guangdong and Fujian provinces [1] and as one of the “Four Southern Herbal Medicines”. It is a plant that likes warm and humid climate, occurs in hillside or hill, mainly at altitudes 200–700 m. The Gaoliang region in Guangdong is the center of cultivation of MO, where the population diversity is preserved well and it has formed abundant cultivars with different phenotypic characteristics and high yield due to the plentiful rainfall, warm climatic and unique geological conditions. Its roots are extensive used as a traditional Chinese medicine to treat many diseases, [2–5], and also used as a functional food owing to its active compounds, such as, oligosaccharides, polysaccharides, iridoid glycosides, anthraquinones, and others [6, 7]. Modern pharmacological studies have shown that oligosaccharides purified from MO have significant effects, including anti-depression, anti-osteoporosis, anti-fatigue and improving immunity. It also has been widely used in the treatment of moderate and mild depression, osteoporosis as well as some geriatric diseases such as Alzheimer’s disease [8–12]. The Chinese pharmacopoeia stipulates that the content of nystose in MO should not be less than 2% [13].

The collection and research of germplasm resources is a vital pillar to initiate breeding programs. The wild resources of MO play an important role in scientific studies and application since they have mainly experienced natural selection and are minimally influenced by artificial selection, however, they are on the verge of extinction at present. Most of MO are cultivated artificially for many years and have been propagated asexually mainly by cuttings, which may get easily infected with *Fusarium* wilt and causes serious degradation of the species. The diversity of germplasm provides plant breeders with the opportunity to develop new varieties and improved them with excellent traits, and is also the basis for limiting genetic erosion. Previous studies have shown that there were significant differences in the appearance traits of different germplasm resources of MO, as well as the chemical components, for example, the leaves of MO from Guangdong province were leathery, with protrusions, pubescent at both upper and lower surface, with high content of nystose, while leaves from Hainan were obviously hairy, more smooth and with extremely low nystose [14, 15]. Analyzing genetic diversity and population structure is significant to expedite the development on breeding strategies and researches on genetic relationships of MO plants. Molecular markers have become powerful tools for the genetic research of MO populations, including RAPD [16], ITS [17], and ISSR [18]. These studies indicate that the current MO populations are highly genetically diverse, especially in Fujian province. However, the MO populations used in previous studies were either small in sample size or narrowly geographically distributed, including only seven MO producing regions in Guangdong and Fujian provinces. The use of a limited number of parental genotypes may have contributed to the narrow genetic base of the current Chinese MO cultivars. Therefore, it is necessary to search for more diverse breeding materials in China to broaden the genetic background of improved varieties. To our knowledge, the population structure and genetic diversity of MO germplasm have never been examined using SNP (single nucleotide polymorphism) markers from next-generation transcriptome sequencing platforms in previous studies.

SNP, as a representative of the third generation of molecular marker technology, has been widely used in the differentiation and identification of various plant and animal germplasm resources [19–22]. RNA-Seq could reduce the complexity of genome compared with the whole genome sequencing, which provides a more effective strategy to identify sequence variations of expressed genes, due to it can detect a large number of SNPs and effectively label most of the gene variations that may affect phenotypic traits [23–25].

Most researchers regard dispersal speciation and vicariance as the two major modes of the formation of plant and animal geographical patterns, such as the quaternary glacial and interglacial oscillations that have had an important influence on the different lineages evolution since populations of ice sheets isolated in distant refuges [26–28]. Manel et al. [29] indicated that the genetic differentiation that seems to be attributed to natural selection may be the result of distance segregation, which restricts gene flow between the populations, or that of the second contact of populations segregated in glacier refugia [30–36]. Climatic oscillation in the past historic also has had a significant impact on today’s biota, many investigations of phylogeography conducted in recent years have clarified the effect of ancient and modern history on the current distribution patterns and genetic variation of plants and animals [28, 37–40]. The species diversity of MO is generally considered to be the result of geographical speciation, which is supported by Ding et al. [17]. As far as we know, there are few researches in MO have involved the development of molecular markers to address population structure and to classify germplasm. Thus, obtaining genomic differentiation and variation of the MO genotype will be helpful to effectively utilize some valuable germplasm resources in future’s breeding programs, not only in China, but also abroad.

In this study, we have extensively collected 88 germplasm resources of MO from ten populations across Guangdong, Guangxi, Fujian and Hainan provinces. The morphological, biochemical and molecular markers were used to comprehensively study the germplasm variation of MO. The aim was to obtain a complete description of genetic diversity of MO accessions came from different regions in China, discover and utilize various gene and genotype resources, search for excellent MO accessions, pave a way for the breeding of new varieties and protection of germplasm resources.

## Results

### Genetic diversity based on morphological traits

**Fig. 1:**
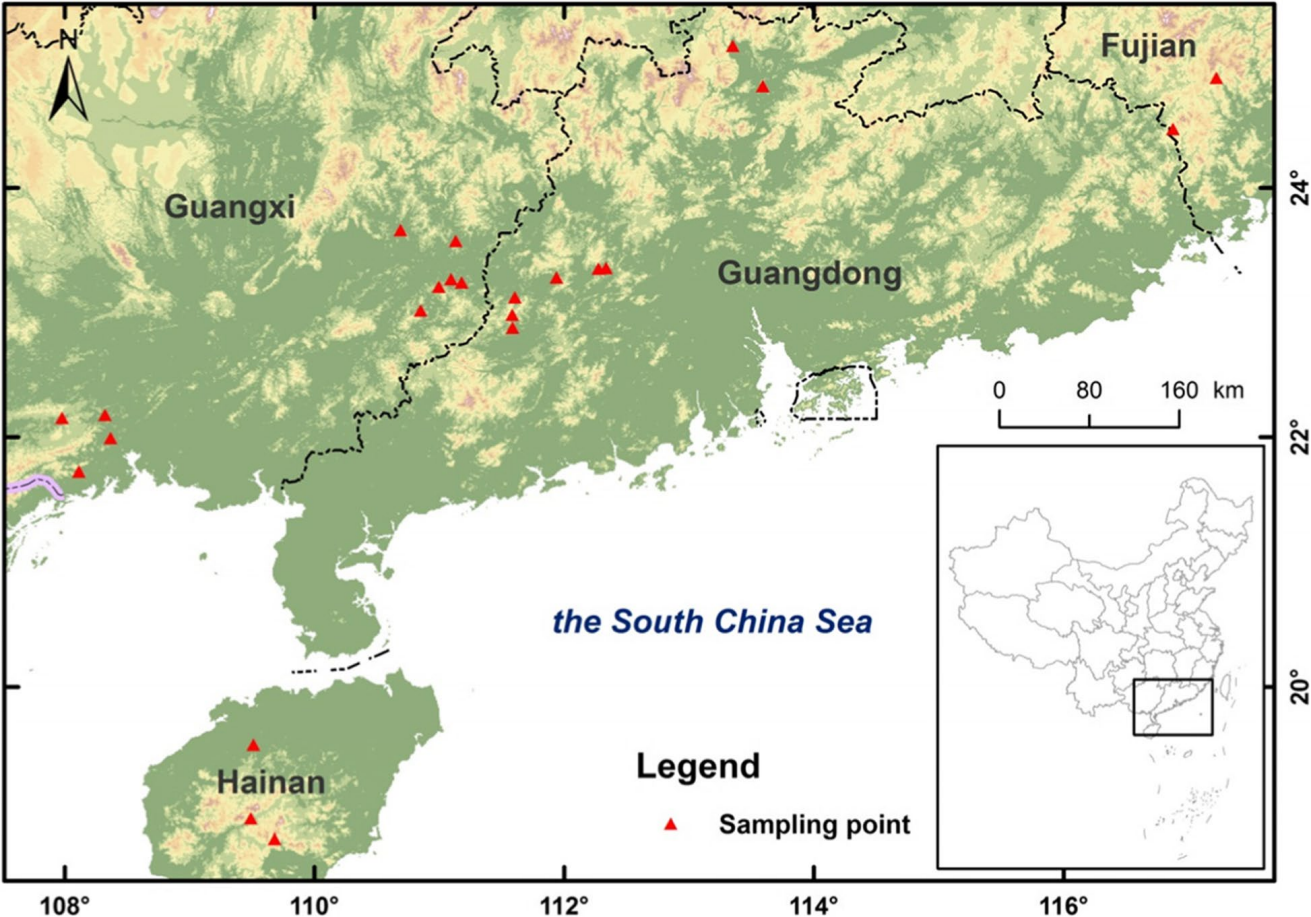
Geographical locations of *Morinda officinalis* in four provinces in China

The geographic distribution of 88 MO populations is shown in Fig. 1. The variation of main morphological characters of MO from different accessions is shown in Table 1 and Table S1. The leaf length ranged from 3.24 cm to 15.51 cm with the maximum of FJ18 (15.51 cm). The leaf width ranged from 1.05 cm to 6.65 cm with the maximum of FJ18 (6.65 cm). The root length ranged from 8.30 cm to104.6 cm with the maximum of FJ12 (104.6 cm). The root diameter ranged from 0.39 cm to 1.54 cm with the maximum of GD28 (1.54 cm). The root range was 7.10 cm ~ 57.50 cm and the maximum was FJ02 (57.50 cm). The number of main roots and lateral roots ranged 1.30 ~ 11.80 and 0.80 ~ 13.00 respectively, and the maximum was FJ12. The section color of the root was 0 ~ 2. The plant height ranged from 7.00 cm to 223.00 cm with the maximum of FJ18(223.00 cm). The results reveal that Fujian samples generally had long root strips, fleshy and thick roots, large root ranges, many branches, good plant growth and lush vines. Guangdong germplasm had short plants and thick roots. Guangxi samples had smaller root strips, shorter plants and narrower leaves. Hainan samples had thin root strips and small leaf area. The Shanon’s diversity index (H′) and coefficient of variation (CV) of the phenotypic traits were calculated for the assessment of genetic diversity of MO accessions. The CV of nine traits ranged from 25.93 to 65.86% with an average of 43.09%. The most variable trait was plant height (65.86%), followed by lateral root number (57.93%), main root number (49.70%) and root length (46.80%), while the root diameter showed least variation (25.93%), indicating that the plant height varied greatly and the root diameter was almost uniform among different accessions. The CV values of the nine phenotypic traits were all above 20%, suggesting that the variability of these traits was obvious.

**Table 1:** Morphological traits of *Morinda officinalis* germplasm utilized in this study

Variables	Max	Min	Mean	SD	CV(/%)	H′
Root length (cm)	104.60	8.30	37.93	17.66	46.56	1.8977
Root diameter (cm)	1.54	0.39	0.82	0.22	26.83	1.9442
Leaf length (cm)	15.51	3.24	7.04	2.15	30.54	1.8807
Leaf width (cm)	6.65	1.05	3.17	1.03	32.49	2.0430
Root range (cm)	57.50	7.10	25.99	9.87	37.98	1.9593
Main root number (piece)	11.80	1.30	3.30	1.64	49.70	1.7275
Lateral root number (piece)	13.00	0.80	3.30	1.93	58.48	1.9273
Plant height (cm)	223.00	7.00	36.07	24.54	68.03	1.4913
Section color of the root	2.00	0.00	1.55	0.59	38.06	0.7930

*SD* Standard deviation, which was calculated based on the measured values of nine traits. CV is an abbreviation of coefficient of variation, which was estimated as the ratio of the standard deviation to the mean of all accessions. H′, Shanon’s diversity index, which is used to investigate the endemic diversity of plant communities

A higher H′ means a higher degree of genetic differentiation. In this study, the H′ of the nine phenotypic traits varied from 0.7930 to 2.0430, with an average of 1.7404. The leaf width (2.0430) and root range (1.9593) were most diverse within the set of MO accessions while the lowest was for the section color. The H′ of other traits was root diameter (1.9442) > lateral root number (1.9273) > root length (1.8977) > leaf length (1.8807) > main root number (1.7275) > plant height (1.4913).

According to the investigation, the accessions of MO in Guangdong province could be divided into three categories: large leaf species, medium leaf species and small leaf species with significantly different phenotypic characteristics. Among them, the large leaf species with yellow-green, elliptic or long-elliptic leaves, which average length was up to 8.5 cm, and the leaves of medium leaf species were dark green, slightly round with an average length of 6.2 cm, while those of small leaf species were dark green and extremely narrow, just like willow leaves, with an average length of 5.5 cm. The number of roots, root length and diameter of small leaf species were larger so that the yield was higher obviously than other germplasms. In addition, we found a kind of modern landrace with purple buds, dark green and lighter leaves, which with more hairs. Most of them were short and the local called “Hei Rui Zai” with larger planting area. However, the same germplasm from different populations varied greatly in morphology, which might be because of the influence of ecological environment and local artificial intervention. For instance, the root length of Hei Rui Zai from Nanjing, Fujian could be as long as 104.60 cm, while that from Wuzhou, Guangxi was only 8.30 cm, and the root was very thin, which might be the result of the backward management level of Wuzhou and the infertile soil.

**Fig. 2:**
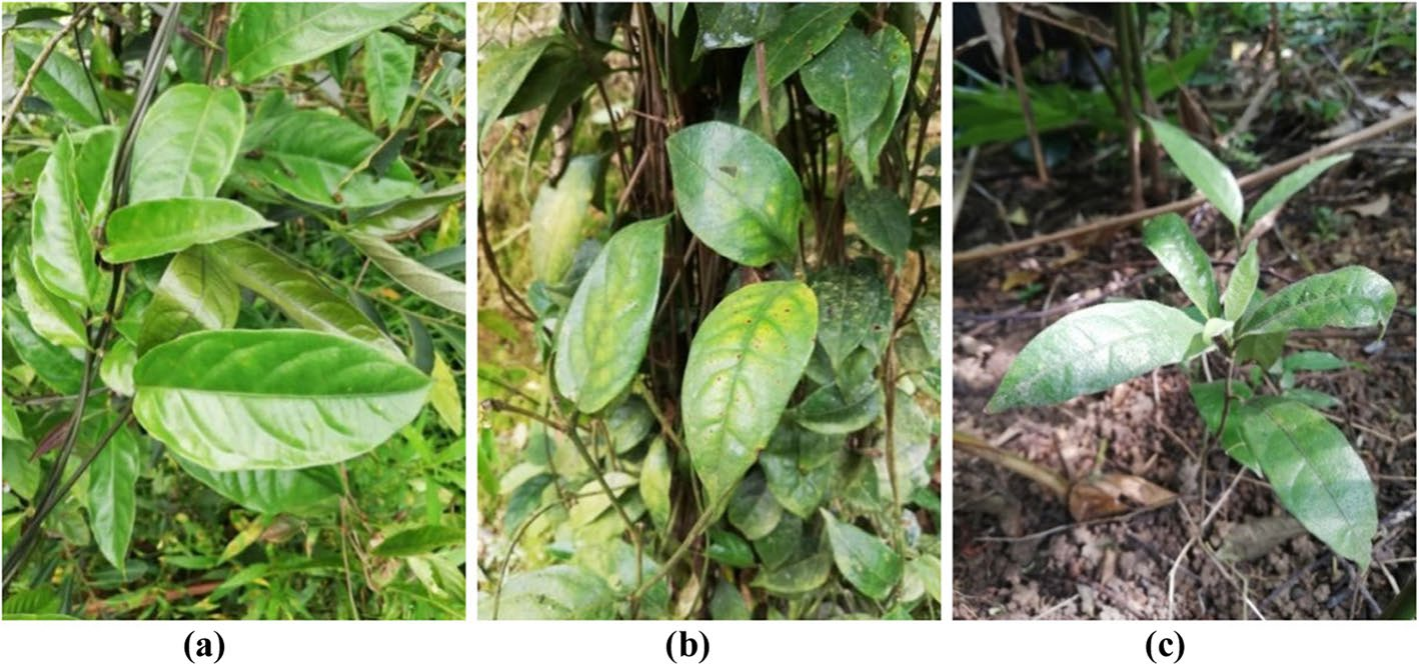
The phenotypic characters of wild species of *Morinda officinalis* in different populations (**a**) Fangcheng; (**b**) Nanjing; (**c**) Danzhou

The wild species from different populations also have large differences in morphology, such as those from Qinzhou and Fangcheng, Guangxi have long spindle shape, dark green, bright surface, less hair leaves, thinner roots, lavender section and longer vines, which wrap in the associated plants. We found a special wild plant in Fangcheng with an average leaf length of 10.3 cm and a leaf width of 4.9 cm, with an unusually bright, glabrous surface and black vines (Fig. 2a). The wild plant from Fujian grows up to 23 years, and the vines are abnormally flourish, as long as 3 ~ 4 m, which are entangled on the associated tangerine trees. The leaf’ shape varied greatly, which is oblong, gray-green with lots of peeling film, and the size in terms of leaf length and width can reach 16 × 8 cm. The roots are thick with an average diameter of 1.7 cm, nodular shape and yellow-white section (Fig. 2b). The wild MO in Hainan is similar in appearance to Guangxi, but the leaves in Hainan are tender green, thin, soft, and papery with the clearly white and smooth hair. The roots are short and generally thin with the mean diameter of 0.574 cm (Fig. 2c). The cross section is unusually gray-black and this might be due to the complex growth environment, which grows under the *Amomum villosum* forest and has a large shade.

### Genetic diversity based on chemical components

The content of oligosaccharides of MO is shown in Table 2 and Table S2. The results show that the content of nystose of MO from different origins varied greatly. The average content of nystose of Guangdong, Guangxi, Fujian and Hainan germplasm was 53.58 mg/g, 51.46 mg/g, 60.42 mg/g and 39.98 mg/g respectively. Fujian germplasm had the highest content of nystose, followed by Guangdong germplasm. We performed the Spearman correlation analysis to explore the relationship among the phenotypic traits, chemical components and geographic position. The results displayed significant and positive correlation between the length and width of leaf, indicating that the longer the leaf length, the closer the blade profile to ellipse (Table S3). The length of root was also positively correlated with root diameter, number of main and lateral root, root range, plant height, the section color and contents of nystose and 1^F^-furanosaccharide nystose, indicating that the feasibility of using these traits to breed new varieties with high active ingredients. However, we found that there was a negative correlation between root diameter and the content of nystose, indicating that the sample with a smaller root diameter has a higher content of nystose, which was in contradiction with the traditional grading standards. Moreover, the phenotypic traits such as leaf width, root length, root range, the main and lateral root number, as well as the content of 1^F^-furanosaccharide nystose were also positively correlated with the latitude and longitude, which suggested that the plantation site with high latitude and longitude is more suitable for the growth and development of MO. Finally, the root length, root range and the contents of oligosaccharides were significantly positively correlated with altitude, which provides scientific basis for the rational cultivation of MO.

**Table 2:** Variation of oligosaccharides contents of *Morinda officinalis* (mg·g^− 1^)

Variables	Max	Min	Mean	SD	CV/%	H′
fructose	65.29	0.00	14.11	13.33	94.47	1.8156
glucose	12.88	0.00	3.27	2.79	85.32	1.8601
sucrose	93.17	15.05	40.43	13.34	33.00	1.9836
1-kestose	77.00	6.97	30.95	13.52	43.68	1.977
nystose	84.37	4.55	55.26	12.76	23.09	2.0427
1^F^-fructofuranosyl nystose nystose	129.25	4,70	79.87	19.94	24.97	2.0485

**Fig. 3:**
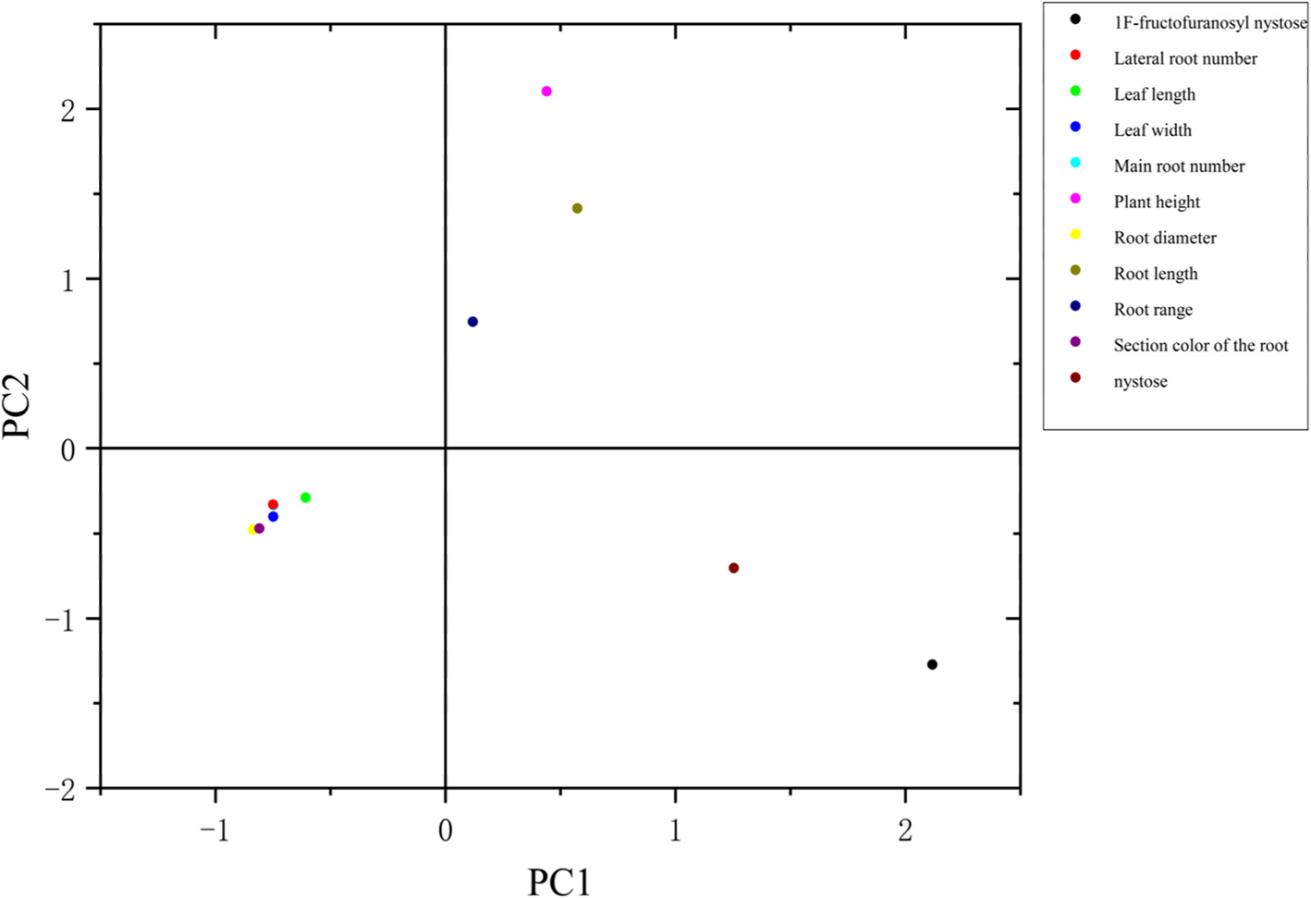
Principal component analysis of variables

Furthermore, PCA analysis revealed that the first four principal components explained 78.889% of the total diversity (Fig. 3 and Table S4). The PC1 with eigenvalue of 3.180 accounted for 28.909% of contribution to total variation with traits such as root range and length. The variables including leaf length and width contributed significantly to PC2, explaining 22.128% of the total variation. The PC3 accounted for 18.027% of contribution to the total variation with variables such as nystose and 1^F^-furanosaccharide nystose. The section color contributed much to the PC4, which explained 9.825% of the total variation. All the 11 variables were slightly differentiated and it was found that they could be used to discriminate the MO accessions.

Assessment of MO genetic diversity using nine phenotypic traits and two main chemical components revealed that 80 accessions were grouped into three distinct clusters (Fig. S1 and Table S5). The cluster I,II and III comprised of 29, 47 and 4 MO germplasms, respectively. The clusterIcontained most samples of Fujian, which had stubby roots, oblong leaves, purple cross section, and relatively less oligosaccharides. Cluster II accounted for the maximum number of materials, which was relatively dwarf with longer roots, bigger root range, more branches, smaller leaves and the highest content of oligosaccharides. The accessions in cluster III had poor growth, small root strips, small root range, long fusiform leaves, and contentand the lowest oligosaccharides and all of them were wild accessions sampled from Guangxi, Fujian and Hainan province. On the whole, considerable variation existed among different populations, and the genetic relationship between them had some correlation with geographic origin, that is, the accessions from the same population were mostly clustered in the same branch, and a few were scattered with other populations. Samples from Guangdong were mostly clustered with those from Fujian, while wild species from Guangxi were closely related to those from Hainan. However, according to field investigations, the samples clustered in the same branch still have large differences in phenotype and the content of chemical components, such as HN01, GX21, GX13, and FJ18 are all wild species and clustered in the same branch, but the leaves of HN01 are thin and covered with soft hairs, while the FJ18 leaves are unusually large and thick with film shedding, this might be because the phenotype is susceptible to environmental factors. Therefore, in order to more accurately classify MO germplasms and reasonably develop and use the resources, it should be further analysis combined with the genotypes of the samples.

### SNP detection

**Fig. 4:**
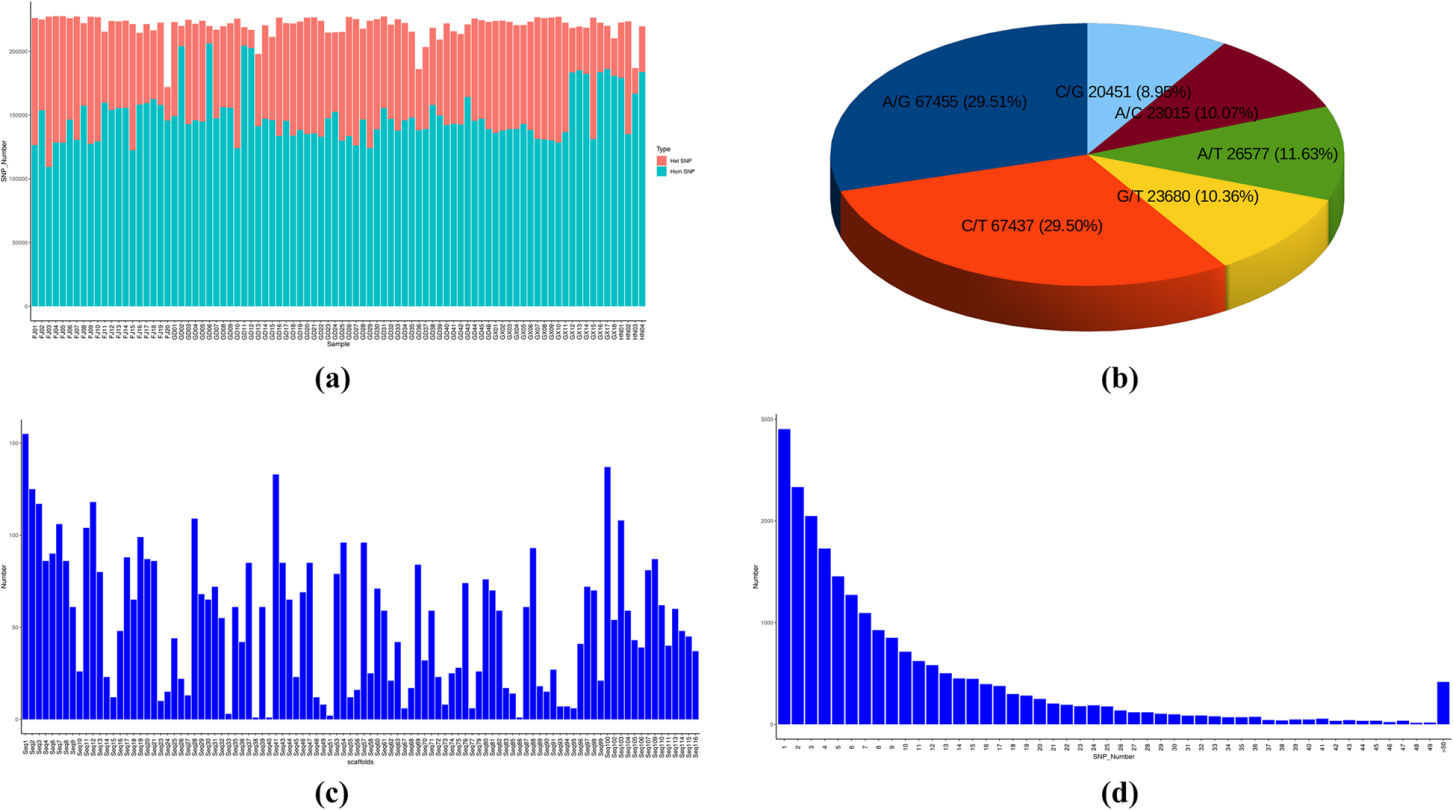
The number and distribution of SNPs. **a** The number of SNPs in 88 *Morinda officinalis* samples. **b** The mutation types of all SNPs. **c** The SNPs’ number among 100 longest scaffolds. **d** The distribution density of SNPs on scaffolds

Putative SNP markers were identified in 88 MO accessions using GATK software. A total of 719,010 SNP markers were included across all libraries when the identified SNPs of all 88 MO samples were combined together, and 228,615 markers were considered good enough to estimate the genetic diversity and population structure of MO after those failing minor allele frequency test (MAF < 0.05) (490,395) were removed. The number of SNPs per sample ranged from 171,984 to 227,6322 with an average of 220,056, and the sample with the largest number of SNPs was FJ05, while the FJ20 with the smallest number. A total of 13,076,979 homozygous SNPs and 6,287,963 heterozygous SNPs were detected in all 88 accessions, accounting for 67.53 and 32.47% of the total number of SNPs, respectively. The average heterozygous rate was 32.26% (Fig. 4a and Table S6), which was found that significantly higher in cultivated samples (33.26%) than that in wild samples (20.78%).

In the SNP dataset, transitions (C/T, 29.50% and A/G, 29.51%) and transversion (C/A, 10.07%; G/T, 10.36%; C/G, 8.95% and A/T, 11.63%) were the two most abundant types of substitution, including 134,892 (59%) and 93,723 (41%) SNPs, respectively (Fig. 4b). The observed transition occurred much more frequent than transversion, with a ratio of 1.44:1, which was similar to the ratio of coverage and mark density. Moreover, we analyzed the SNPs’ distribution across all scaffolds in the MO nuclear genome, and the results showed that 228,615 SNPs were distributed on 13,905 scaffolds and 22,383 transcriptome genes, respectively. The number of SNPs on 100 longest scaffolds were counted and it was found that Seq1 contained the most SNPs, while the Seq38、Seq40 and Seq86 harboring least SNPs (Fig. 4c). Further analysis revealed that approximately 3000 scaffolds containing only one SNP out of 13,905 scaffolds with SNPs (Fig. 4d). Those suggesting that the SNPs in this study have a relatively high coverage in the genome and a uniform distribution, which can be effectively used to analyze the genetic diversity of MO accessions.

All of 228,615 SNPs were divided into several categories based on their positions in the genome (Table 3). Out of the total putative SNPs, 176,005 (76.78%) were identified in genetic region while the other 53,078 (23.22%) in intergenic region. A total of 36,842 (16.12%) SNPs were identified, resulting in synonymous variants. In particular, there were 49,509 (21.66%) SNPs classified as non-synonymous SNPs, which caused changes in the coding amino acids and affected the function of the coding genes, leading to changes in biological traits. The other 182 and 286 SNPs gave rise to mutations in start and stop codons, respectively, which led to changes in the protein transcription process, resulting in the loss of protein function, and ultimately affected the phenotype of the species. The remainder caused variants located in 3′ UTR and 5′ UTR, and they were not involved in coding.

**Table 3:** Distribution of SNPs from different regions of *Morinda officinalis* transcriptome

Regions	Substitution types	Number of SNPs	Proportion (%)
Genetic region	Synonymous	36,842	16.12
	Non-synonymous	49,509	21.66
	Start codon variant	182	0.08
	Stop codon variant	286	0.13
	5’UTR	31,327	13.70
	3’UTR	57,859	25.31
Intergenic region	Intergenic region	53,078	23.22

**Fig. 5:**
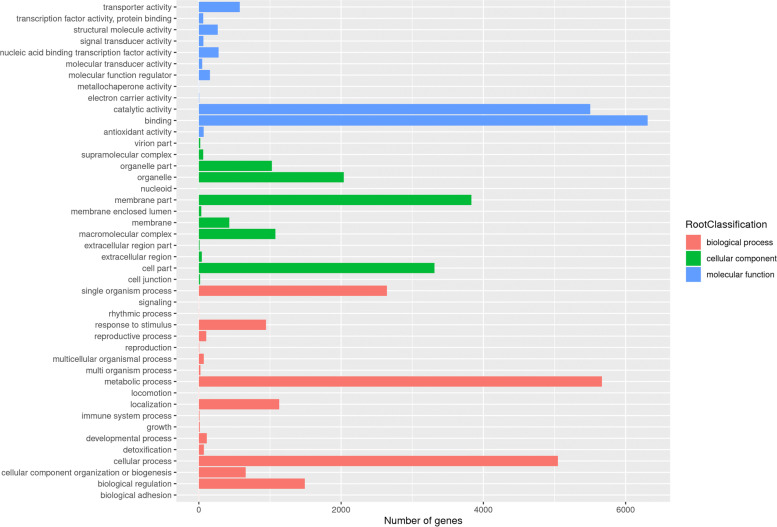
GO enrichment of genes with non-synonymous SNPs in *Morinda officinalis*

Gene ontology (GO) classification was used to distinguish the available functions of the genes with non-synonymous SNPs or other SNPs that alter amino acid sequence based on sequence homology. A total of 43,202 genes were categorized into three main GO terms: biological processes, molecular function and cellular components (Fig. 5), and they were then sorted into 45 second classification terms. Within the biological processes, metabolic, cellular, and single organism process were the most highly predominant terms, while the biological adhesion and signaling term included only one gene. For the molecular function category, binding, and catalytic activity were the most abundant terms. And the membrane part, cell part and organelle were the most highly represented GO terms regarding the cellular components category. Furthermore, there were 5666 genes were classified into the metabolic process terms, indicating that they were closely related to metabolic pathways.

### Genetic diversity analysis of *Morinda officinalis* germplasm resources

A total of 192 SNPs that were all detected in MO samples, which were selected from 228,615 validated SNP cites, and these genotypes obtained were all population specific (Table 4). The heterozygosity can reflect the genetic variation of the population in multiple locus and it is considered to be an optimal parameter for measuring the genetic diversity of the population. The observed heterozygosity (Ho) and expected heterozygosity (He) ranged from 0.16 to 0.46, and 0.17 to 0.37 with an average of 0.34 and 0.29, respectively, suggesting that MO populations were less affected by outside selection, inbreeding and other factors, and was in a state of genetic balance. The highest value of observed number of alleles (Na) was found in the Nanjing population (1.97), while the Danzhou (1.25) showed the smallest value. The effective number of alleles (Ne) ranged from 1.25 in the Qinzhou and Danzhou populations to 1.62 in the Nanjing population. The Shannon’s information index (I) varied from 0.17 (Danzhou) to 0.53 (Nanjing) with a mean of 0.37, suggesting that the community diversity of Nanjing was relatively high. The highest (0.36) and lowest (0.13) Nei’s genetic distance (Nei) were identified in the populations of Nanjing and Danzhou, respectively. The percentage of polymorphic bands (PPB) ranged from 25.00% (Danzhou) to 97.40% (Nanjing), and the average was 66.87%. The global F_IS_, F_IT_ and F_ST_ values were calculated by STRUCTURE software to evaluate the population structure level. The F_IS_ and F_IT_ were ranged from − 0.97 to 0.87 and − 0.97 to 0.95, with the mean of − 0.40 and − 0.07, respectively. The higher value of F_IS_, which was measured at the subpopulation level of the entire population indicates that the populations lack heterozygosity and high specificity. The F_ST_ varied from 0 to 0.72 with an average of 0.24 (Table S7), suggesting that there was a large genetic differentiation and a low degree of inbreeding among populations. The gene flow (Nm) estimated from F_ST_ varied from 0.10 to 91.56 and the average of which was recorded at 0.80 (Table S7), indicating that MO subpopulations have a certain frequency of gene flow. The exchange of genetic material can reduce genetic drift in subpopulations, and can avoid the strong differentiation between subpopulations to a certain extent. The distribution of the minor allele frequency (MAF) was analyzed, as shown in Fig. S2. The MAF ranged from 0.05 to 0.1 with the most SNP loci (about 50,000), followed by 0.1 to 0.15, indicating that the MO populations involved in this study have rich genetic diversity.

**Table 4:** Summary statistics of molecular diversity revealed by SNP markers in ten *Morinda officinalis* populations from four provinces in China

Population	Sample size	Na	Ne	I	Ho	He	Nei	PPB
Deqing	23	1.8646	1.4526	0.3892	0.3610	0.2632	0.2574	86.46%
Shaoguan	2	1.6562	1.5125	0.4079	0.4609	0.3776	0.2832	65.62%
Yunan	10	1.7656	1.4892	0.4083	0.4318	0.2902	0.2757	76.56%
Gaoyao	11	1.8906	1.5361	0.4699	0.3854	0.3290	0.3140	89.06%
Wuzhou	11	1.4948	1.4569	0.3272	0.4389	0.2447	0.2335	49.48%
Fangcheng	4	1.7708	1.4293	0.3983	0.2669	0.2993	0.2619	77.08%
Qinzhou	3	1.3646	1.2477	0.2093	0.1649	0.1705	0.1421	36.46%
Nanjing	20	1.974	1.6163	0.5282	0.4161	0.3651	0.3560	97.40%
Danzhou	1	1.2500	1.2500	0.1733	0.2500	0.2500	0.1250	25.00%
Wuzhishan	3	1.6562	1.4085	0.3623	0.2604	0.291	0.2425	65.62%
Mean	8.8	1.6687	1.4399	0.3679	0.3436	0.2881	0.2491	66.87%

*Na* Observed number of alleles, *Ne* Effective number of alleles, *I* Shannon’s information index, *Ho* Observed heterozygosity, *He* Expected heterozygosity, *Nei* Nei’s genetic distance, *PPB* Percentage of polymorphic bands

Through model-based analysis, the analysis of molecular variance (AMOVA) was carried out to estimate the population genetic constitution using its reliability and consistency, which can provide some valuable information (Table 5). The result revealed that the majority of genetic variation (99.66%; Df = 166; sum squares = 82.40) occurred among individuals within populations, whereas 0.58% of the variation (Df = 6; sum squares = 3.22) was detected among populations within groups and the remaining came from among-groups. AMOVA showed that the genetic variation of MO germplasm mainly originates from individuals.

**Table 5:** Analysis of molecular variance (AMOVA) in 88 *Morinda officinalis* based on SNP loci

Source	Df	Sum of squares	Variance components	Percentage of variation
Among groups	3	1.542	−0.0012	− 0.24
Among populations within groups	6	3.216	0.00290	0.58
Among individuals within populations	166	82.401	0.49639	99.66
Total	175	87.159	0.49809	100

The genetic distances (GD) and genetic identity (GI) between ten populations were calculated based on the data of SNP locis, and the results showed that the genetic distances between Qinzhou and Danzhou and other populations were larger, which ranged from 0.0556 to 0.3229 with an average of 0.2269, while those between other populations were relatively smaller, only distributed between 0.0073 and 0.2915, with an average of 0.0968 (Table S8). It revealed that there was a high degree of genetic relationship among the subpopulations of MO except Qinzhou and Danzhou, which was consistent with the results of investigation of plant morphological characters. Among them, the maximum genetic distance was observed between Qinzhou and Wuzhou (0.3229), and the smallest was between Deqing and Yunan (0.0073). The genetic identity varied from 0.7240 to 0.9927, with an average of 0.8747. The trend of which was opposite to the genetic distance.

### Phylogenetic analysis of *Morinda officinalis* population

**Fig. 6:**
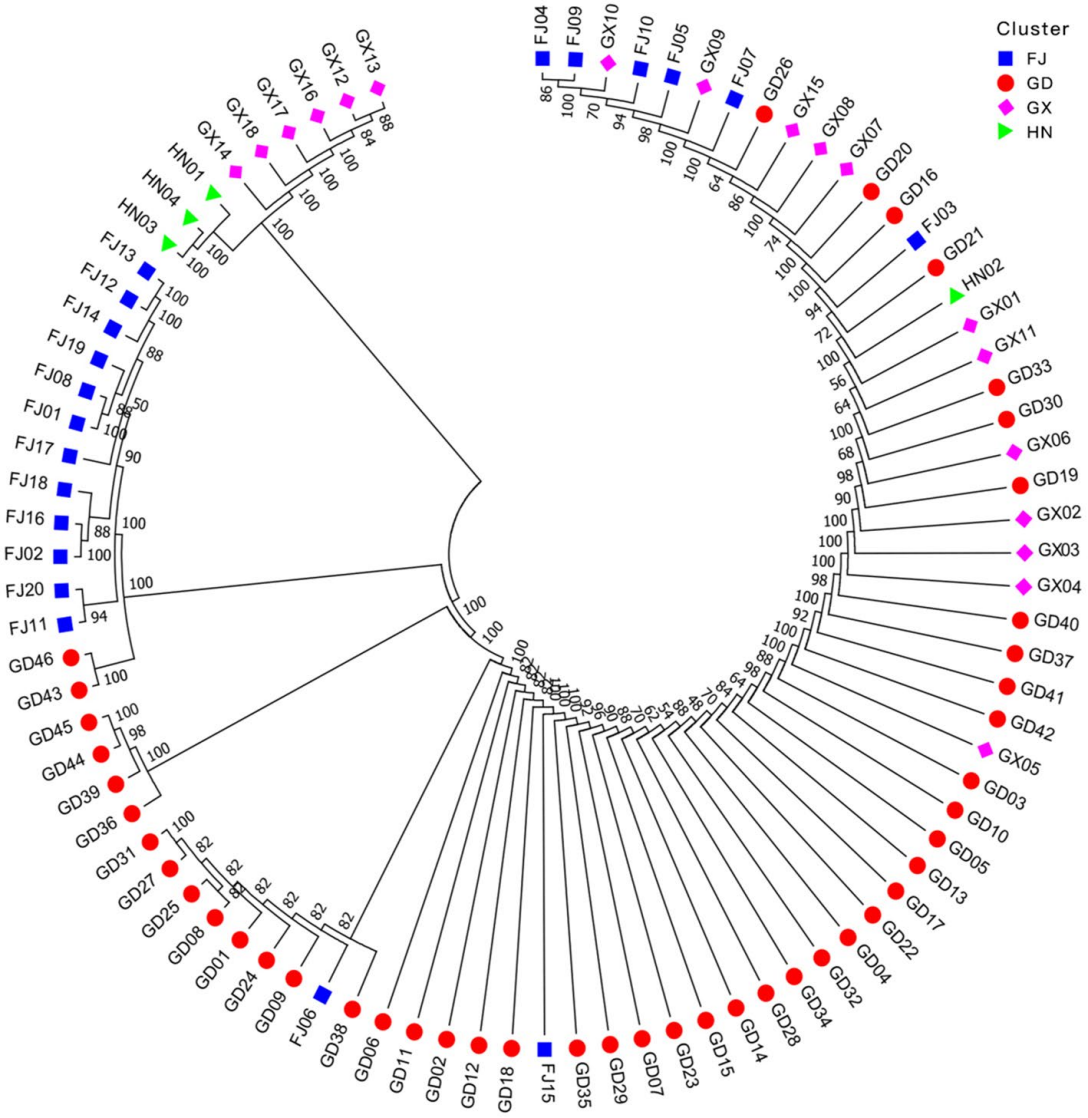
Primary population structure and UPGMA clustering for 88 *Morinda officinalis* accessions

The unweighted pair group method using arithmetic average (UPGMA) phylogenetic tree was generated based on the genetic identity, and the result revealed that 88 MO accessions were mainly grouping in three clusters (Fig. 6). The clusterIcontained nine wild accessions of Guangxi and Hainan, which was dwarf with slender roots, smallest root range, lest branches, and the lest content of oligosaccharides (Table S9). In this cluster, the highest genetic distance (GD = 0.375) was observed between GX12 and HN01, while the lowest was observed between the GX13 and HN01 (GD = 0.071), all of them had rich genetic diversity and could be distinguished from cultivars obviously, which agreement with the phenotypic traits. The wild accessions from Guangxi and Hainan were closely related to each other, suggesting that they could be used to broaden the genetic background. The cluster II included 14 accessions from Fujian and Guangdong, all of them were breeding lines except FJ18 and the genetic distance among them ranged from 0.054 to 0.393. On the contrary, they had stubby and long roots, biggest root range, most branches, and the most content of oligosaccharides. A total of 65 accessions were grouped in the cluster III, most of them were produced in Guangdong province and they were cultivars apart from GX11. In this clade, the genetic distance varied from 0 to 0.643, and some accessions had minimal genetic distance such as GD09 and FJ10, therefore they were considered as closely related to each other. On the whole, the genetic distances between MO accessions had a greater relationship with geographical origin, that is, most of MO germplasms from the same or similar origin could be gathered together, and a few test materials mixed into other groups. The accessions from Fujian were closely related to those from Guangdong and Guangxi, while the wild types from Guangxi were closer to those from Hainan, which was similar to the results of cluster analysis based on phenotypic traits.

### Structure analysis of *Morinda officinalis* population

The mixed Bayesian clustering model-based method in STRUCTURE analysis was used to determine the group of population. The results suggested that K (the randomly mating subgroups’ number) was equal to 3 as the optimal number of groups (Fig. S3a), indicating that the 88 accessions could be classified into three subpopulations (I, II and III) (Fig. S3b). The genotypes of different populations were divided into pure and mixed types according to the sharing of genomic regions, that is, the genotypes with the score of ≥0.80 were considered as a pure group and divided into corresponding subgroups, while those < 0.80 were regarded as admixture. Out of the 88 accessions, 29 were admixtures (approximately 33%) and 59 were pure (approximately 67%). In the admixtures, 52% were collected from Fujian and 48% from Guangdong, contained only one wild type (named as‘Admixed Wild Type’), which indicated that the genetic background of genotypes from Fujian and Guangdong was more complex, with mixed genes from multiple populations and higher level of genetic diversity, while those from Guangxi and Hainan were pure type with narrower genetic background. Above results were consistent with those of UPGMA analysis. In addition, most of the wild accessions were pure type (named as‘Pure Wild Type’) such as GX13and GX14, which might be the results of their relatively isolated growth environment and could be used for MO breeding.

### Principal component analysis of *Morinda officinalis* population

**Fig. 7:**
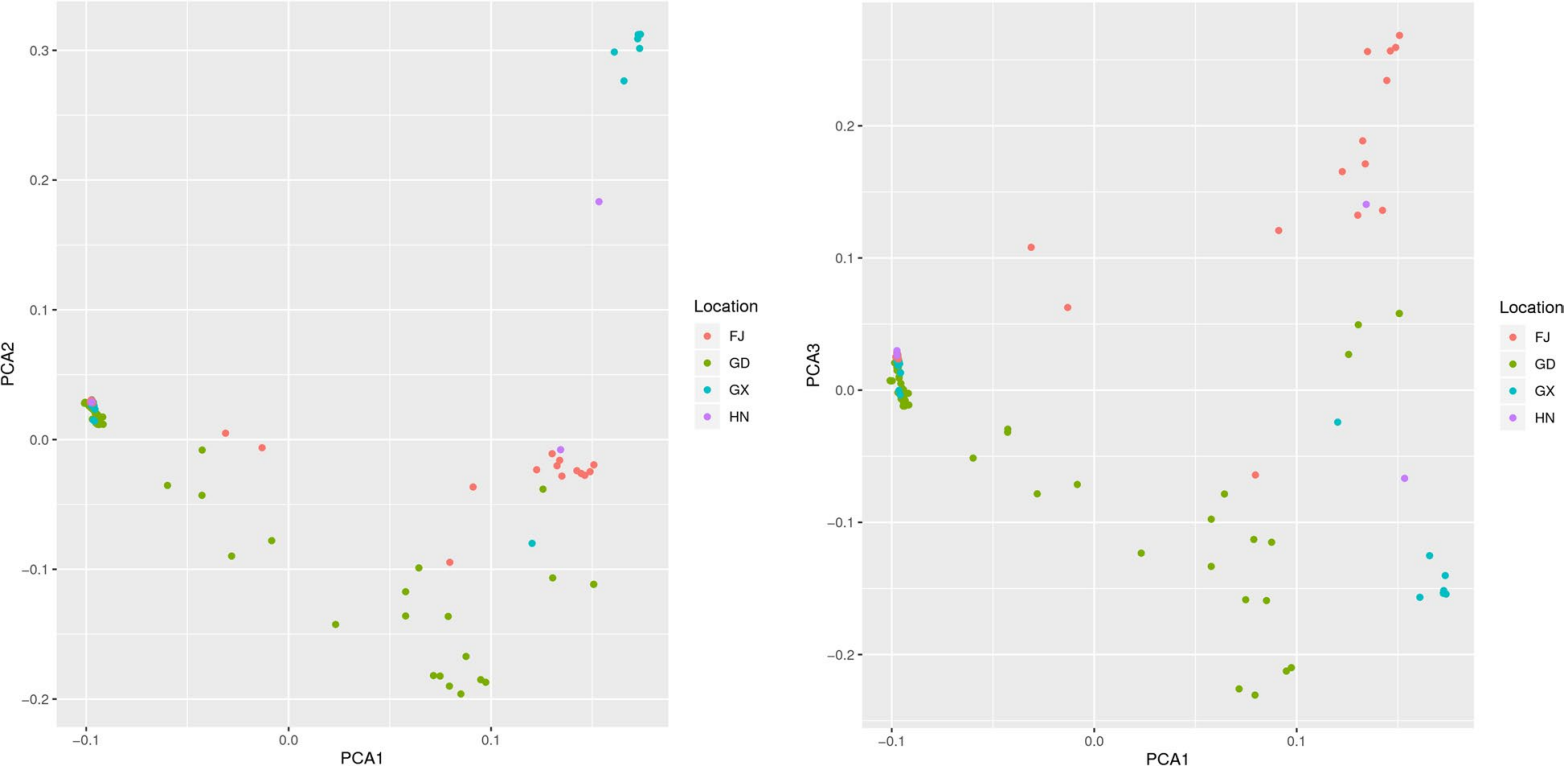
Principal component analysis (PCA) of 88 *Morinda officinalis* accessions

The result of PCA analysis was in agreement with those of population structure analysis, which showed three distinct clusters. As shown in Fig. 7, the accessions from Fujian were more scattered than those from Guangdong, followed by Guangxi and Hainan. The Guangxi accessions were basically clustered together except GX12 and GX14, which clustered with HN03 and HN02, indicating that the genetic differentiation of genotypes from Guangxi was slight and there was low variation among the germplasms. A few scattered germplasms might have been introduced from other regions.

## Discussion

Genetic diversity refers to the sum of genetic variation among individuals within a species or within a population [41, 42]. The study of genetic diversity can not only provide information on the level of population genetic structure, but also serve as a platform for selecting superior genotypes as parents in medicinal plants improvement breeding programs [43, 44]. In this study, we analyzed the genetic diversity of the cultivated and wild MO samples based on phenotype, chemical composition and genotype. The results showed that there exists abundant genetic variation in the MO populations. There are great genetic differentiations in phenotype among different varieties, such as large leaf species with long and large leaves, while small leaf species with extremely narrow leaves, and the same varieties from different populations also have certain differences, as well as different geographical origins. Phenotypic differentiation of traits might be the result of a genetic divergence due to the genetic drift, local adaptation, as well as gene flow restriction [45, 46]. MO is a perennial vine with a long generation cycle and is androgynous [17], which mainly transmitted by pollen under natural conditions, which may affect the flow of genes to a certain extent. The cross-pollination characteristics of MO species may also contributed to the high level of genetic variation in the populations. In previous study, we found a counterfeit species of MO (locally named “Bajigong”) in the field investigation, which had larger leaves, thinner phloem and thicker wood core. And the plant *Morinda citrifolia* of the same genus is a common adulterant of MO due to the similar appearance traits, whether MO can hybridize with pollen of these plants to generate different agricultural types remains to be further studied.

Modern pharmacological studies revealed that oligosaccharides with polymerization degree of 3–7 have obvious antidepressant effects. Therefore, in this study, nystose, 1^F^-fructofuranosyl nystose were combined with 9 phenotypic traits and geographical locations for correlation analysis, and it was found that the content of nystose in MO was negatively correlated with root diameter, but in actual production, it is often believed that samples with larger root diameters have better quality thus have higher prices. The results of this study are in contradiction with the traditional classification criteria, which found that the finer the roots, the higher the content of active ingredients, especially near the root tips. This pattern is also observed in other medicinal plants such as *Glycyrrhiza uralensis* [47] and *Arabidopsis thaliana* [48], possibly because of the high auxin content near the root cap. MO accessions from high altitude and latitude have a high content of nystose, which is consistent with the growth habits of traditional MO, and it might be that the plantation at higher altitudes has plenty rainfall and sufficient sunlight, which is beneficial the growth of MO and the accumulation of active ingredients. This result provides a reference for growing high-quality MO. The results revealed that several phenotypic traits were correlated with the content of active components, such as the length of root. Therefore, we can use OTL identification and genome-wide association study (GWAS) to associate the traits of interest with SNP markers, and develop SNP sites that control phenotypic traits for breeding research. In addition, it is reported that phenotype-related genes are more likely to be synonymous rather than non-synonymous SNP, because synonymous SNP may affect the secondary structure of RNA and cause allelic imbalance, altering gene expression, thereby influence the phenotypic trait [49]. Synonymous play an important role in phenotype affecting, and focusing only on non-synonymous SNP in molecular researches will lead to many functional variations being ignored.

To our knowledge, breeding practice has a greater impact on reducing the level of genetic diversity compared with domestication, resulting in lower genetic diversity in cultivars than that in wild varieties [50–52]. In this study, we found that the wild types of MO were significantly different from cultivated types not only in morphological traits, chemical compositions, but also in genotypes. This probably due to there exist some ancient landraces in cultivars, which originate from the early landraces or their natural offspring, and had a certain impact on the genetic differentiation of MO populations. The relatively isolated ecological environment of wild types might reduce the genetic interference from other MO varieties. A narrow genetic diversity of cultivars not only limited the breeding process of MO, but also increased the risk of natural disasters [53, 54]. The wild type plants and landraces could provide valuable genetic resources for breeding programs, both them could be used for the breeding of new MO varieties to broaden genetic background.

The higher the heterozygosity rate, the more similar the base sequence between two DNAs, and the closer the relationship between them [55]. The heterozygous rate of MO (average heterozygous rate = 32.26%) is significantly higher than other species, such as rice [56], soybeans [57], etc. That’s probably because these crops have undergone manual selection for a long time and the degree of genetic diversity has been reduced, while the MO planting through cutting propagation for many years with mixed seedings, and so that the genetic basis is complex. Interestingly, we observed a significantly higher heterozygosity in the cultivars compared with the wild types, which is inconsistent with those reported in other species [58, 59]. An explanation can be considered for this counterintuitive finding: MO is propagated generation by generation, and the variation gradually accumulates, while the wild species are in a relatively isolated environment for a long time with the smaller genetic variation. These are just our preliminary conclusions about genetic variation among wild populations due to the limited number of wild individuals per population analyzed in this study, and more diverse populations need to be collected for further analysis.

Phylogenetic and population structure analysis results showed that there was a significant and positive correlation between gene exchange and geographical distance, revealing an isolated pattern by distance within the distribution of MO, further confirming the limited gene flow between populations and facilitating the local adaptability establishment. The genetic distance between MO accessions from Fujian, Guangdong and Guangxi was relatively close, and the wild species in Guangxi were closely related to those in Hainan. It is speculated that the evolution process might be that the ancestral population of Guangdong area was more suitable for MO growth due to the local environment and climate, so that the local breeding was relatively fast, and then spread to Fujian, Guangxi, and Hainan. Guangxi might have expanded from Fujian and Guangdong. However, since the geographical barrier of Qiongzhou Strait there was little gene exchange between MO populations in Hainan and Guangdong province, as well as Fujian [17]. In addition, we found that the local expansion and variation of the populations were obvious in Hainan, this probably attributed to the great influence of its geography and unique climate. Furthermore, wind and animals have long been considered as the primary agents of inter-population gene exchange between geographically isolated populations, which can transport pollen and seeds to other MO plantations over the landscape, and can reduce the impact of genetic drift and founder events [60]. Since the gene flow that wind mediated appears to be effective just at distances of less than 1 km, anemochorous dispersal may only affect allele frequencies among closer populations, while birds can transport pollen and seeds over 12 km, and long-distance migration promotes gene flow between local populations, making the populations genetically homogeneous [55, 61]. These are consistent with the results of field investigation: the plant morphological characteristics of Hainan population have significant variation, followed by Guangxi, while the plants of Guangdong and Fujian were similar.

The low similarity was observed in this study between the phylogenetic analysis based on phenotypic, chemical components and SNP markers, it might be because the phenotypic traits are under influence of environment and genotype, any phenotypic traits could be as the outcome of one or combination of few mutations and thus the genetic distance between different germplasm is quite different [62–64]. Therefore, it might not be a reliable approach to access of genetic diversity of MO populations based solely on morphological traits. DNA markers such as SNP and SSR are routinely used to analyze the genetic diversity of the genotypes since they rely on a large number of mutations throughout the entire genome and can provide important information at the genetic level, regardless of environmental factors [65–67]. The low correlation between two methods might also be that phenotypic traits are often affected by natural and artificial selection, while the variations detected at the gene level are usually of the non-adaptive types and are therefore not susceptible to selection [45]. Analysis of population structure and genetic diversity based on accessions’ phenotypic traits is more convenient, but genotypic analysis is relatively accurate, and the two methods complement each other. Therefore, several authors point out that the best way is to combine the two methods to identify genetic differences [45, 68], which can further our understanding of the genetic background of the population due to the abundant and non-overlapping information.

The high proportion of admixtures could be recognized as an indication of gene change in MO populations [69, 70]. A lot of accessions in this study were derived from hybrid lines with genotypes of different genetic backgrounds, which enhanced the level of genetic diversity. Population structure analysis revealed that GD24, GD01, GD38, GD09, GD27, FJ03, FJ15, FJ06 and FJ20 were admixtures, indicating that they have a high degree of genetic differentiation and the presence of gene flow in the MO populations. These samples may have some excellent traits that other germplasms absent or disappeared, such as disease resistance, cold resistance, high yield, etc., and they can be further utilized as parent materials for cross breeding programs and broaden the genetic basis of MO. These materials are very important for the breeding of new varieties of MO, therefore, it is necessary to protect their natural habitats in order to protect the overall genetic diversity within population. Species conservation mainly focused on protecting of genetic diversity and evolutionary potential, and its strategy must be based on the understanding of population genetic structure. The following measures are recommended for protection: Priority should be given to protecting endangered populations. Moreover, since the high degree of genetic differentiation between populations, the loss of genetic diversity in any population will lead to the reduction of genetic variation, it is therefore crucial to protect all populations in place, restore the size of all groups on this basis, protect their habitats and promote their renewal. Ex situ strategies could be considered to avoid inbreeding decline, and accessions should be sampled according to the genetic structure of natural population to prevent genetic homogenization of cultivated population [66]. Also, a germplasm bank could be established to protect the genetic diversity of different groups of the species. Last but not least, genetic analysis should be introduced into further breeding programs to facilitate the hybridization and introduction of cultivation and sustainable development of the MO varieties.

## Materials and methods

### Plant materials

Eighty-eight MO accessions representing diverse origin were collected from ten populations in Guangdong, Guangxi, Fujian and Hainan province of China (Table S10). Forty-six accessions came from Guangdong province, eighteen from Guangxi, twenty from Fujian while the rest originated from Hainan province. The samples were identified by Prof. Ping Ding (Guangzhou University of Chinese Medicine, Guangdong, China). A voucher specimen (No.20180625046) was deposited in School of Pharmaceutical Sciences, Guangzhou University of Chinese Medicine. These local genotypes were considered diverse across regions, as each region gradually selected individuals that adapt to the environment and traditional characteristics of the origin area. Therefore, the 88 genotypes selected may represent most of the genetic diversity observed in Chinese MO populations. Each plant was divided into two parts, one for morphology and chemical composition analysis, the other was used to isolate fresh root tip tissue and immediately frozen in liquid nitrogen and stored at − 80 °C until RNA extraction.

### Evaluation for morphological traits

Morphological evaluation was carried out by investigating 9 important and genetically stable phenotype traits as follows: root length, root diameter, leaf length, leaf width, main root number, lateral root number, plant height, plant width and section color. The vernier caliper and band tape were used to measure the phenotypic traits of MO accessions, 10 biological replicates and 3 technical replicates were selected for each material. In order to facilitate subsequent analysis, the section color was assigned, purple was denoted as “0”, lavender was denoted as “1”, and light yellow was denoted as “2”.

### Oligosaccharide extraction and HPLC-ELSD analysis

Chromatographic analysis was performed using an HPLC (Primaide-1110, JPN) equipped with an ELSD-UM5800 detector. The column was Waters XBridge™ Amide (4.6 mm × 250 mm, 3.5 μm, Techway, USA). The mobile phase consisted of 0.2% triethylamine acetonitrile (A) -0.2% triethylamine water (B), and the linear gradient was set as follows: 0–10 min for 75% A to 70% A, 10–20 min for 70% A, 20–45 min for 70% A to 60% A, 45–60 min for 60% A, and 60–63 min for 60% A to 75% A. The following parameters were also set: volume flow of 0.8 mL·min^− 1^, sample injection volume of 20 μL, column temperature of 35 °C, ELSD drift tube temperature of 75 °C, and nitrogen flow of 2.5 L ·min^−^ 1 [71].

Standards of oligosaccharide (i.e., fructose, glucose, sucrose, 1-kestose, nystose and 1^F^-fructofuranosyl nystose) were purchased from Shanghai Yuanye Biotechnology Co., LTD (Shanghai, China). The lot numbers of standards are SS0905GA14, SA0418GA14, TF0226CA14, C09D8Q50061, Z17A9H59088, S09A8D41431, respectively, and the purity is greater than 98%. The samples were crushed, and 0.5 g of each sample was soaked in 50 mL of 50% ethanol solution. Ultrasonic extraction was performed for 20 min after half an hour of rest, and then centrifuged at 3000 rpm for 10 min. The upper layer was collected, filtered through at 0.22 μm microporous membrane filtration and then transferred for HPLC-ELSD analysis [71].

### RNA extraction and sequencing

Total RNA from each sample was extracted using the RNA prepPure Plant Kit (Tiangen, Beijing, China) following the manufacturer’s instructions. The quality and concentration of RNA samples were assessed with a Nanodrop spectrophotometer (Thermo Scientifc, USA) and the integrity of RNA was determined using Agilent 2100 Bioanalyzer.

The high-quality RNAs were used for cDNA library construction. Oligo (dT) labeled magnetic beads were used to combine the polyA of the mRNA for purifying the mRNA. The mRNA was fragmented with the size of 200–300 bp at elevated temperature in the presence of divalent cations and the first-strand cDNA is synthesized, and this cDNA was transformed into double-strand cDNA using DNA polymerase I and RnaseH. The cDNA was cleaned by AgencourtAmpure XP SPRI beads (Beckman-Coulter). The protruding termini of the DNA fragments were end-repaired and added “A” base, and adapters were ligated to the cDNA fragments. The sequencing library was constructed using 10 cycles of PCR. The concentration and size of the cDNA library were determined using Qubit 2.0 Fluorometer (Invitrogen, Carlsbad, CA, US) and quantified with Agilent 2100 Bioanalyzer (Agilent Technologies, Santa Clara, CA, USA). Furthermore, all libraries were sequenced using the Illumina HiSeq 4000 platform with a paired-end reads length of 150 bp at Science Corporation of Gene (Guangzhou, China) [71].

### SNP calling and annotation

After the sequencing of the Illumina paired-end, raw reads were filtered by removal of adapter sequences, reads containing poly-Ns, and reads with a low-quality score (*Q* < 20). The high quality reads were then de novo assembled into contigs and transcripts using the Trinity package (version 2.4.0) with the default parameters. The transcripts were clustered to reduce data redundancy by blasting against the nr protein database and the longest sequences in each cluster were reserved as unigenes.

The raw reads of 88 MO accessions were aligned to the reference genome sequence using the STAR software (version 2.7.1a) with default parameters [72]. The picard tool (v2.0.1) was used to add the sample information. The generation of the GTF files and quantification of the genes or isoforms were carried out from the mapping BAM files using StringTie (version 1.3.3b) tool. Filtering the mapping results by Reassigning Mapping Qualities, Split “N” Trimming and Duplicate markings. SNPs calling were performed using the HaplotypeCaller in the GATK variant pipeline with a minimum phred-scaled confidence threshold of 30 from the genome-mapped alignments. The filter parameters of SNP were set as QD < 2.0, FS > 60.0 to obtain high quality SNPs. The functional effects of the SNPs were annotated and predicted using the program SnpEff (version 4.3 k) based on their positions in genomic, which provides a simple estimate of the impact of mutation, this is, high (add or remove stop codons, frame shifts, etc.), moderate (change, delete or inserte codons, etc.) and low (synonymous changes, etc.) [73]. Finally, in order to eliminate putative sequencing errors, the minor allele frequency (MAF) < 0.05 SNPs were removed, and only the SNPs with the most informative were retained for downstream analysis.

A Gene Ontology (GO) analysis was conducted to annotate the genes harboring non-synonymous SNPs with the MO nuclear genome used as background references. The AgriGO web tool was used to identify enriched GO terms and those with corrected-pvalue ≤0.05 were considered significantly enriched by the hypergeometric test.

### Population genetics analysis

Genetic diversity of the population is generally estimated by parameters, such as Shannon-Weaver (*H′*) index, Minor allele frequency (MAF), Observed heterozygosity (Ho), Expected heterozygosity (He), Observed number of alleles (Na), effective number of alleles (Ne), Nei’s gene diversity index (Nei), Shannon’s information index (I), The percentage of polymorphic bands (PPB), Fixity coefficient (F_st_), Gene flow (Nm), Genetic identity (GI) and Genetic diversity (GD), they were estimated by PowerMarker 3.25 and Popgene version 1.32 software [74]. Among them, *H′*is a basic index to measure the genetic diversity of population and was calculated as follows:

$${H}^{\prime }=-\sum P_{\mathrm{i}}\mathrm{ln}P_{\mathrm{i}}$$

where *H′*is Shannon-Weaver index, *P*_i_ is the frequency of the i^th^ variant type.

The variation coefficient could be calculated using the following equation:

$$cv=\frac{\upsigma}{\upmu}$$

where *σ* represents the standard deviation, and *μ* represents the arithmetic mean, which is used to measure the degree of dispersion of per trait.

In order to describe the variance components of MO individuals and population differentiation among ten assumed subgroups, AMOVA was carried out using GeneAlEx 6.502 program with 1000 permutations [75].

The population structure of MO was analyzed using the Bayesian model-based method implemented in STRUCTURE 2.3.4, employing the assuming admixture model with correlated allele frequency. The optimal number of populations present within the 88 accessions was identified using ten independent runs and setting the K from one to ten. The parameters were used as follows: a burn-in period of 100,000 with Monte Carlo Markov China (MCMC) iterations of 100,000. The number of true clusters in the data (K) was determined by the CLUMPAK software. And the optimal K (the number of subpopulations) was estimated based on the posterior probability of the data for a given K, as well as the △K. Finally, the full search algorithm was used to align the replication results of the cluster analysis.

To offer an additional support for the population structure and the number of populations of MO accessions, this study also carried out the PCA using Genome-wide Complex Trait Analysis (GCTA) software with default parameters [76]. In order to analysis the genetic relationships among samples in this study, the UPGMA tree was performed using TASSEL v.5.2.37 [77] software with a simple matching coefficient.

## Supplementary Information

**Additional file 1.**

**Additional file 2.**

**Additional file 3.**

## Data Availability

RNA sequence data were deposited in NCBI under the BioProject accession number PRJNA848112. The results of SNP calling and annotation are presented in table SA in Additional File 1.
