# Supplementary material for: Phenotypic, chemical component and molecular assessment of genetic diversity and population structure of Morinda officinalis germplasm
Source: BMC Genomics. 2022 Aug 19;23:605. doi: 10.1186/s12864-022-08817-w (PMC9392303; doi:10.1186/s12864-022-08817-w)
Supplement: Supplementary file 3 — Additional file 3. [file 12864_2022_8817_MOESM3_ESM.docx]

**Phenotypic, chemical component and molecular assessment of genetic diversity and population structure of** ***Morinda officinalis* germplasm**

Zhenhua Luo^1^, Zien Chen^1^, Mengyun Liu^1^, Li Yang^1^, Zhimin Zhao^2^, Depo Yang^2^ and Ping Ding^1*^

^1^School of Pharmaceutical Sciences, Guangzhou University of Chinese Medicine, Guangzhou 510006, China

^2^School of Pharmacy, Sun Yat-sen University, Guangzhou 510006, China

^*^ Correspondence: Ping Ding

dingping@gzucm.edu.cn


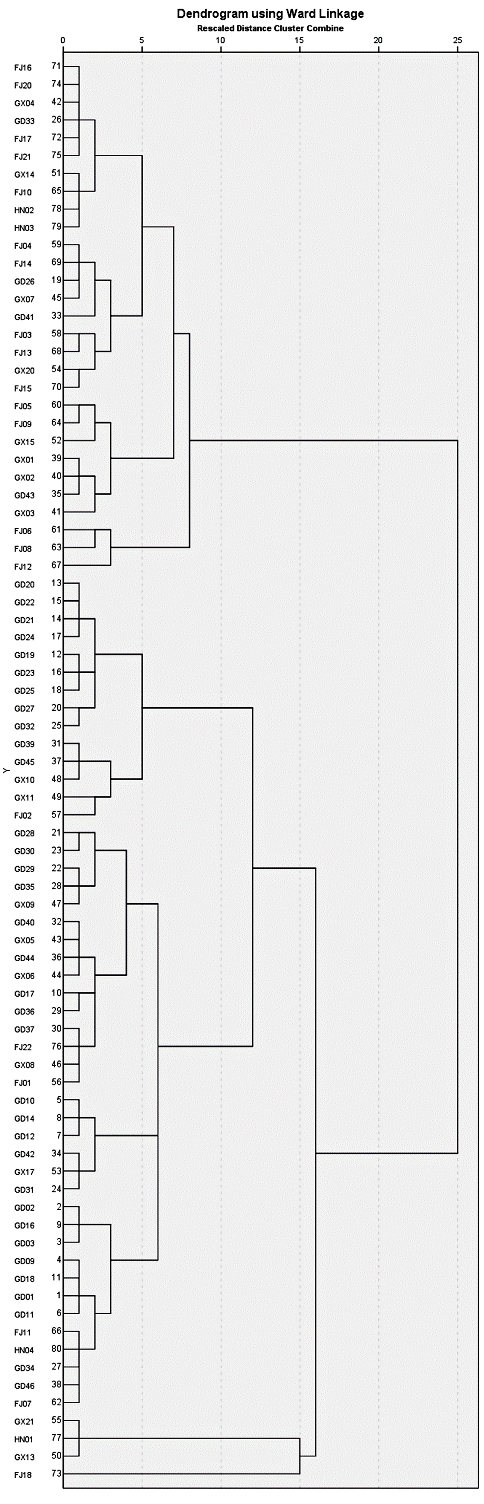


**Fig. S1** Cluster analysis of phenotypic traits and oligosaccharide contents in the studied accessions of *Morinda officinalis*


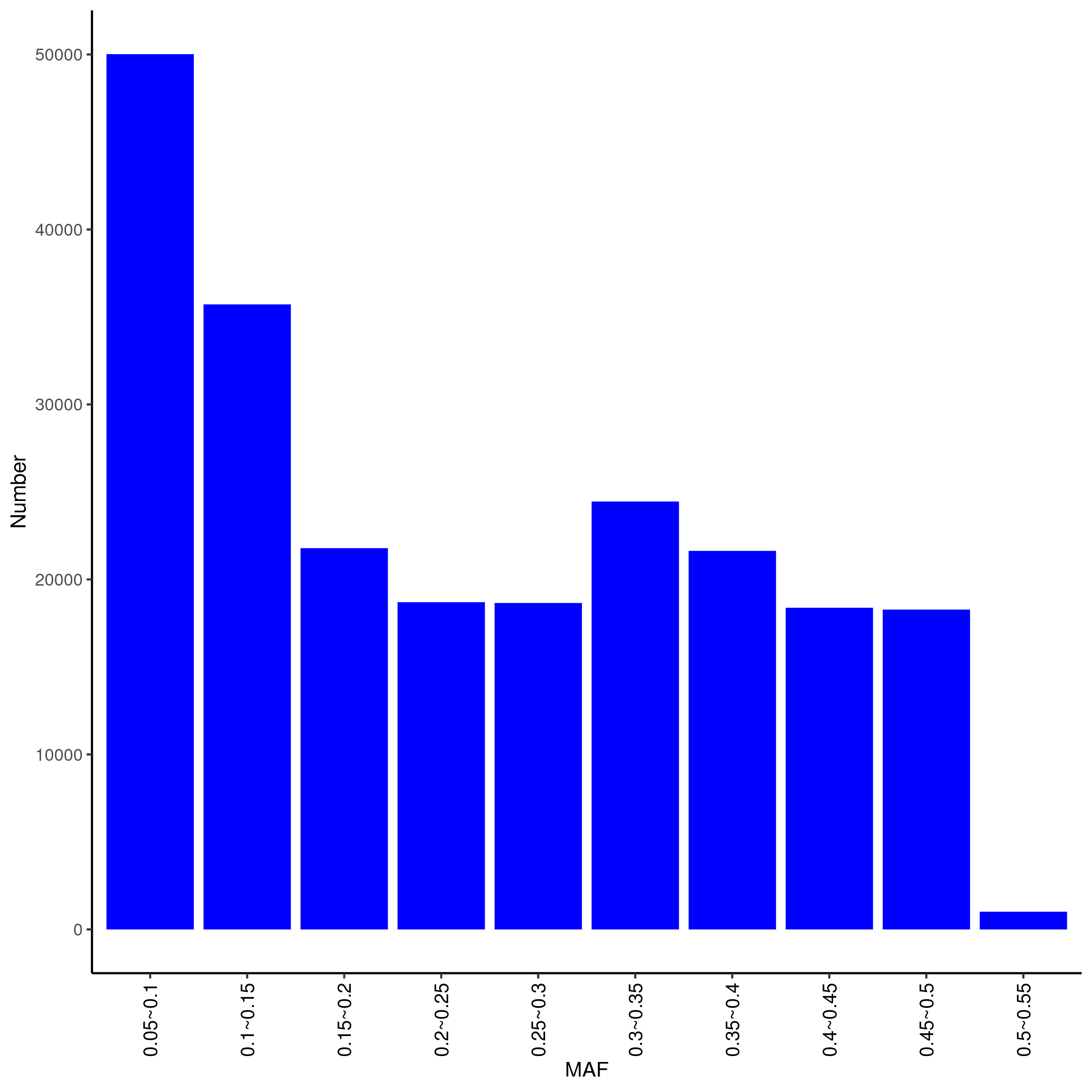


**Fig.S2** The distribution of minimum allele frequency (MAF) of SNPs in 88 *Morinda officinalis* genotypes


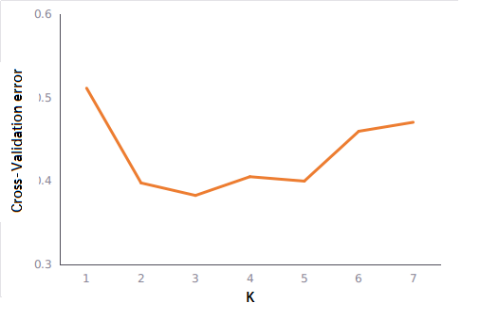

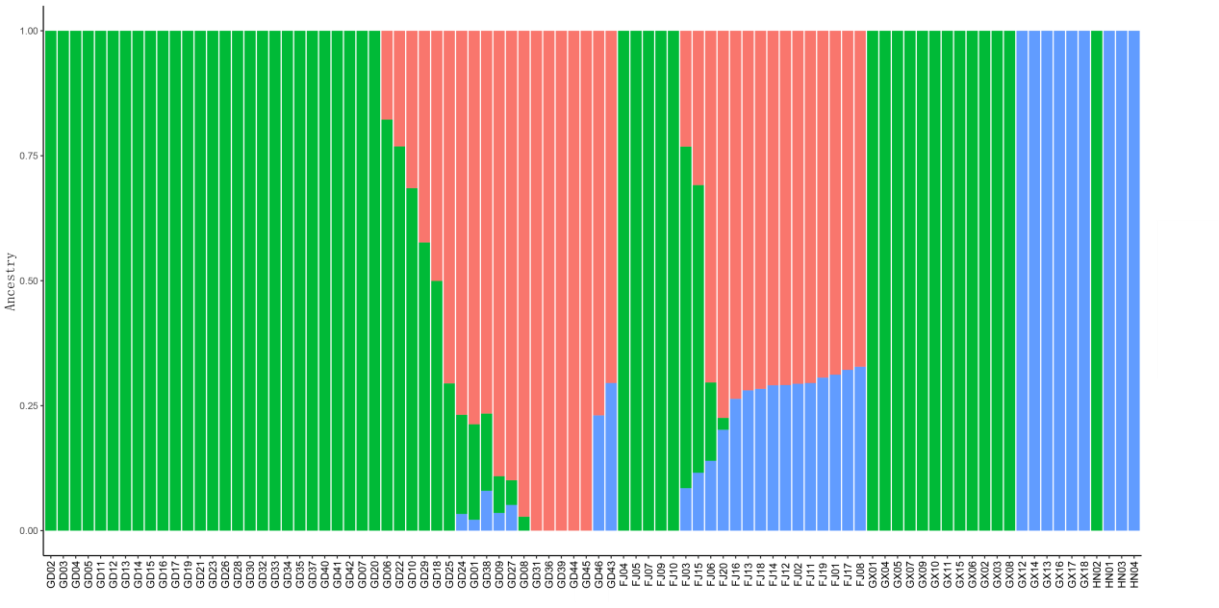


**（a）**

**（b）**

**Fig. S3 (a)** Population structure analysis on 88 *Morinda officinalis* accessions using ADMIXTURE. Estimated cross-validation error of possible clusters (K) from 1 to 7;**(b)** Population structure of the *Morinda officinalis* accessions based on K=3. The 88 MO materials were divided into three subgroups by STRUCTURE, each group were indicated by different colors
